# Imported malaria in Rio de Janeiro state between 2007 and 2015: an epidemiologic approach

**DOI:** 10.1590/0074-02760190064

**Published:** 2019-06-13

**Authors:** Hermano Gomes Albuquerque, Paulo Cesar Peiter, Luciano Medeiros Toledo, Paulo Chagastelles Sabroza, Rafael dos Santos Pereira, Jefferson Pereira Caldas, Jussara Rafael Angelo, Cristina Giordano Dias, Martha Cecília Suárez-Mutis

**Affiliations:** 1 Fundação Oswaldo Cruz-Fiocruz, Instituto Oswaldo Cruz, Laboratório de Doenças Parasitárias, Rio de Janeiro, RJ, Brasil; 2 Fundação Oswaldo Cruz-Fiocruz, Escola Nacional de Saúde Pública Sérgio Arouca, Laboratório de Monitoramento Epidemiológico de Grandes Empreendimentos, Rio de Janeiro, RJ, Brasil; 3 Secretaria de Estado de Saúde do Rio de Janeiro, Rio de Janeiro, RJ, Brasil

**Keywords:** malaria, imported infectious diseases, epidemiology

## Abstract

Imported malaria is a malaria infection diagnosed outside the area where it was acquired and is induced by human migration and mobility. This retrospective study was performed based on secondary data from 2007 to 2015. In total, 736 cases of imported malaria (79.7% of 923 cases) were recorded in Rio de Janeiro state. Of the imported cases, 55.3% came from abroad, while 44.7% came from other regions of Brazil. Most cases of imported malaria in Brazil (85.5%) originated in Amazônia Legal, and Burundi (Africa) accounted for 59% of the cases from abroad. Analyses of the determinants of imported malaria in Rio de Janeiro state must be continued to understand the relationship between the origin and destination of cases.

Malaria is one of the most important infectious diseases worldwide, and eliminating it has been on the global agenda.^1^ However, the spread of malaria worldwide is favoured by migration, which causes disease spread to nonendemic areas or areas where malaria was previously eliminated.^2^ Currently, in Brazil, the Amazon Rainforest is a malaria-endemic region.^3^ Cases recorded outside of the Amazon are called extra-Amazonian malaria cases, and they can be autochthonous (if acquired by local transmission) or imported. The latter is defined as a malaria infection acquired outside the area where it was diagnosed.^4^

We created a conceptual model of malaria for the extra-Amazon Region based on receptivity and vulnerability criteria.^5^ In our model, we consider that migration (migratory or mobility) could be a vulnerability factor for imported malaria in the extra-Amazon Region.

Imported cases represented most of the Brazilian extra-Amazonian malaria cases between 2007 and 2014, with 5,416 recorded cases (88.9% of all cases) and only 676 autochthonous cases.^6^ The fatality rate owing to malaria in this region was 60 times higher than that in the Amazon,^3^ mainly because of the late diagnosis and treatment of patients, as health professionals in nonendemic areas lack the expertise to handle such cases.^6^ During a dengue epidemic, this context tended to be worse.^7^

Malaria is reported in all states in the Brazilian Southeast region, namely São Paulo, Espírito Santo, Minas Gerais and Rio de Janeiro.^8^ ^,^ ^9^ In these states, except Espírito Santo, imported malaria cases are more frequent than autochthonous cases. In Rio de Janeiro state, 808 cases were detected from 2002 to 2010, of which 773 (95.6% of the total) were imported cases.^9^ Recently, Rio de Janeiro state notably had an outbreak of malaria caused by *Plasmodium simium*.^10^

Regarding the elimination of malaria in Rio de Janeiro state, understanding the receptivity of the territory and the population’s vulnerability to malaria is key for strengthening the elimination agenda. *Anopheles darlingi* (Root, 1926), previously the main malaria vector in this region, was recently found in Rio de Janeiro^11^ where there are other secondary vectors, which demonstrates the receptivity of this territory.^12^ Therefore, this study aimed to advance the understanding of the epidemiology of imported malaria in Rio de Janeiro state as a vulnerability factor.

Rio de Janeiro has 92 municipalities, with an estimated population by Brazilian Institute of Geography and Statistics (IBGE) of 17,159,960 in 2018. Its territory covers 43,780 km^2^. One of Brazil’s major ports, which is the third busiest international airport and the seventh busiest regional airport, is in Rio de Janeiro state; it is well connected to its neighbouring states by wide highways, allowing huge migration, which is an important factor affecting the dynamics of imported malaria.

This retrospective study reviewed data from the Epidemiological Surveillance Program of the Health Service in Rio de Janeiro. The data refer to cases from 2007 to 2015 and included the number of malaria cases, parasite species, municipality of residence, probable geographic site where patient was infected, classification (autochthonous or imported), sex, and age. Patients’ identifying information was not used. However, not all cases confirmed by diagnosis were included because some relevant information necessary for the analyses was missing. The study was approved by the Fiocruz Ethical Committee (process 30606614.2.0000.5248). Descriptive statistical analyses were performed using SPSS 23.0 Statistical Software (SPSS Inc., Chicago, MI, USA) and ArcGIS Desktop 10.0 (Environmental Systems Research Institute, Redlands, CA, USA).

From 2007 to 2015, 736 cases of imported malaria (79.7% of total malaria cases) were reported in Rio de Janeiro state by the Health Department’s Epidemiological Surveillance Program (Fig. 1). The survey reported the highest number of imported cases in 2012 (131/736, 17.8%) and 2011 (90/736, 12.2%), with fewer imported cases in 2015 (61/736, 8.3%) and 2014 (56/736, 7.6%). Besides these imported cases, we identified 69 autochthonous cases in Rio de Janeiro state, and 118 cases had missing data for this outcome (total of 923 malaria cases).

**Fig. 1:**
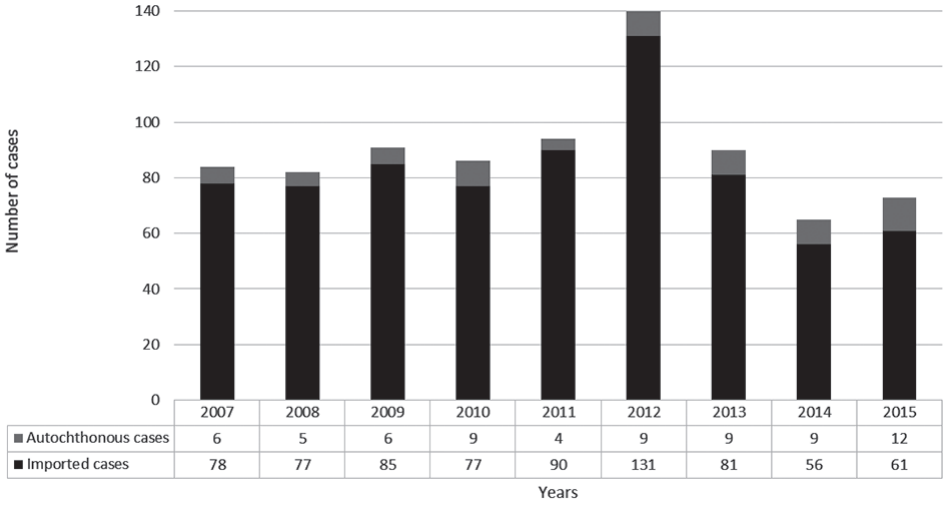
imported malaria cases and percentage per year in Rio de Ja neiro from 2007 to 2015. Font: Epidemiological Surveillance Program of the Health Service of Rio de Janeiro.

Patients were predominantly men (n = 565/736, 76.5%), with women representing approximately one-fourth of cases (n = 171/736, 23.2%). This pattern has been the same over the years with low variability. Patient age ranged from 1 to 85 years (mean, 36.7 ± 13.6; median, 36.0). During the study period, “traveller” was the main activity category of the individuals with imported malaria (n = 266/736, 36.1%), and “tourism” was the second main activity with 44 cases (6% of the total).

The origin of the imported cases showed that 384 (55.3% of the total) came from abroad, while 352 (44.7%) came from other regions of Brazil (Fig. 2). Internationally imported cases came mainly from Central Africa (n = 244/736, 33.1%), America (n = 33/736, 4.4%), and West Africa (n = 26/736, 3.5%). Notably, 227 cases (30.8%) came exclusively from Burundi in Central Africa. For Brazilian cases, Amazonas state was the main place of origin for malaria (n = 126/736, 17.1% of total), followed by Rondônia (n = 64/736, 8.6%) and Pará (n = 40/736, 5.4%). The states with the lowest number of malaria cases were Minas Gerais (n = 1), Piauí (n = 1), Paraná (n = 1), and São Paulo (n = 1). Amazônia Legal accounted for 85.5% (301 cases) of the national imported malaria cases in the Rio de Janeiro state.

**Fig. 2:**
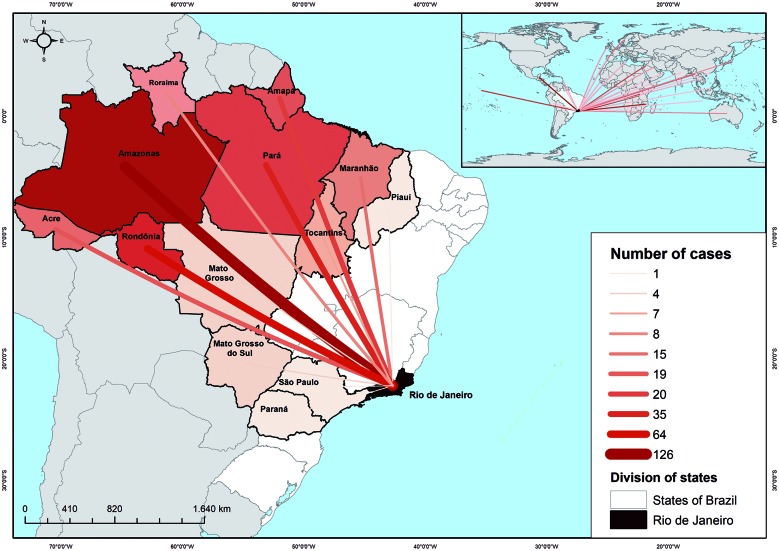
origin state of imported malaria cases in Rio de Janeiro state from 2007 to 2015.

The origin of Brazilian imported malaria cases was reported from 78 different municipalities of Rio de Janeiro state. Five municipalities accounted for 43.7% of the total cases in Brazil. These five municipalities were Manaus (Amazonas state, n = 81/736, 11%), Porto Velho (Rondônia state, n = 43/736, 5.8%), São Luis (Maranhão state, n = 10/736, 1.3%), Belém (Pará state, n = 10/736, 1.3%), and São Gabriel da Cachoeira (Amazonas state, n = 10/736, 1.3%). Moreover, 592 patients with imported malaria were living in Rio de Janeiro state, of whom 402 (68%) lived in the city of Rio de Janeiro, the state capital. The remaining 190 cases were distributed among 41 municipalities throughout the state, with the highest number of diagnosed cases in Macaé (n = 25/736, 4.2%), Niterói (n = 23/736, 3.8%), São Gonçalo (n = 17/736, 2.8%), and Duque de Caxias (n = 17/736, 2.8%).

Excluding the 65 cases (8.8% of total) with missing data, the most prevalent species in thick smear diagnosis was *Plasmodium falciparum*, found in 368 samples (54.8% of all imported malaria cases). The proportion of all *P. falciparum* cases from abroad was almost 80%. The second most prevalent species was *P. vivax*, detected in 279 tests (41.5% of total). Moreover, 15 patients had mixed malaria (*P. vivax* and *P*. *falciparum*; 2.2% of the total).

The yearly analysis of imported malaria cases exhibits two tendencies through the years (Fig. 1). First, from 2007 to 2012, we had a regular trend of cases with a median of 81.5 cases per year, where the highest number of cases was reported in 2012, with almost 50% more cases recorded than that in the second highest year. After 2012, a downward trend was observed in 2014 and 2015, with less than half of the cases reported in 2012. Until 2012, the trend of imported malaria cases in Rio de Janeiro state was contrary to the declining Brazilian malaria trend.^13^ However, the trend between 2012 and 2015 was decreasing following the national trend of malaria cases.

There was a difference between the trend in imported malaria cases in Rio de Janeiro state and the trend in the extra-Amazon Region,^6^ which was due to the year 2012. This could be attributed to cases from abroad, of which Burundi accounted for 59% of imported malaria cases. Burundi is an African country known for malaria epidemics, with 8.3 million confirmed cases in 2016, mostly caused by *P. falciparum*.^14^ This may explain the proportion of *P. falciparum* in imported malaria cases in Rio de Janeiro state (almost 80% of cases), although autochthonous malaria cases in Brazil were caused by *P. vivax* (88.4%).^15^ Interestingly, Brazil opened the Embassy of Burundi in Brasilia in 2012, which led to the greatest number of bilateral encounters between both countries. Furthermore, since 2007, Brazil participates in events for the Development of Burundi.^16^ Further investigation is needed to determine the flow of people between Brazil and Burundi to understand the distribution of imported malaria cases. Strengthening an agile and efficient diagnostic and treatment system, especially for patients from endemic countries, is a fundamental strategy against severe malaria caused by *P. falciparum*.^3^ ^,^ ^7^

The individual profile of imported malaria follows the classic malaria profile in Brazil.^9^ ^,^ ^17^ ^,^ ^18^ ^,^ ^19^ The occupation of these individuals is another important feature.^20^ We found that 36.1% of imported cases involved travellers, but we did not consider this in our analysis because of missing data.

Cohen et al.^21^ reviewed the resurgence of malaria worldwide. They found 78 events of resurgence from 1930 to 2000, of which 68 cases (91%) were attributed to the weakening of malaria control programs. Resource constraints were a cause in 39 of these 68 cases, (57%). In essence, Brazil was in a large economic and political recession, which led to resource contingency. It created a dangerous scenario in Rio de Janeiro state, which despite the interruption of malaria transmission in the past, remains receptive to malaria in some areas, with environmental conditions conducive to vector reproduction and malaria transmission.^5^ ^,^ ^22^ Recently, Espírito Santo had a malaria outbreak of 138 confirmed cases and one confirmed death probably because of an imported case, which shows that imported malaria cases can be a major public health problem.^23^ ^,^ ^24^

The determinants of imported malaria in Rio de Janeiro state must be further analysed to understand the relationship between the origin and destination of cases. On that basis, a surveillance system should be structured to improve early detection, provide adequate treatment, or predict chances of outbreaks. Economic and political relationships between countries, which promote development, appear to be an important determinant for migration and mobility and consequently for the spread of imported malaria.^25^ ^,^ ^26^ We need to understand the main kinds of industries, companies, and businesses that create the context of a country’s vulnerability to malaria. The search to find other databases, which may explain the relationship between the origin and destination of patients, or the spread of malaria induced by migration and mobility to Rio de Janeiro state could be a methodology in future investigations. We need to understand the context of malaria transmission and the local situation of patients who live in Rio de Janeiro state. Moreover, the many cases reported in 2012, as well as Burundi being the origin of most cases coming from abroad, must be clarified.

Our results reveal the spatial and temporal heterogeneity of the origin and destination of imported malaria cases. The main determinants behind these patterns should be understood.
